# Platelets are recruited to hepatocellular carcinoma tissues in a CX3CL1‐CX3CR1 dependent manner and induce tumour cell apoptosis

**DOI:** 10.1002/1878-0261.12783

**Published:** 2020-09-02

**Authors:** Shuo Miao, Meng Lu, Yue Liu, Dan Shu, Ying Zhu, Wei Song, Yuanyuan Ma, Rong Ma, Bixiang Zhang, Chao Fang, Zhang‐Yin Ming

**Affiliations:** ^1^ Department of Pharmacology School of Basic Medicine Tongji Medical College of Huazhong University of Science and Technology Wuhan China; ^2^ School of Basic Medicine Qingdao University Qingdao China; ^3^ The Key Laboratory for Drug Target Research and Pharmacodynamic Evaluation of Hubei Province Wuhan China; ^4^ Department of Pharmacy Renmin Hospital Wuhan University Wuhan China; ^5^ Pharmacy Department Union Hospital Tongji Medical College Huazhong University of Science and Technology Wuhan China; ^6^ Department of Surgery Tongji Hospital Tongji Medical College Huazhong University of Science and Technology Wuhan China; ^7^ Tongji‐Rongcheng Center for Biomedicine Huazhong University of Science and Technology Wuhan China

**Keywords:** CX3CL1/CX3CR1, HCC, migration, platelet

## Abstract

The mechanisms and biological functions of migrating platelets in cancer remain largely unknown. Here, we analyzed platelet infiltration in hepatocellular carcinoma. We detected platelet extravasation in both mouse and human HCC tissues. CX3CL1 directly induced platelet migration, and hypoxia enhanced platelet migration by upregulating CX3CL1 expression. Knocking down CX3CL1 in HCC cells reduced platelet migration *in vitro*, as well as infiltration of HCC tissue in an orthotopic HCC mouse model. Components of the CX3CR1/Syk/PI3K pathway were essential for CX3CL1‐induced platelet migration. Migrating platelets induced HCC cell apoptosis *in vitro*, as indicated by a reduced mitochondrial membrane potential and an increased percentage of apoptotic cells. In the orthotopic tumor implantation model, decreased platelet infiltration was associated with accelerated tumor growth. Taken together, our findings indicate that HCC cell‐derived CX3CL1 contributes to tumor infiltration by platelets, which in turn promotes apoptosis of HCC cells.

## Graphical abstract

Here, we detected platelet extravasation from blood vessels in both mouse and human hepatocellular carcinoma (HCC) tissues. We show C‐X3‐C chemokine ligand 1 (CX3CL1) derived from HCC cells induces platelet infiltration through a CX3CR1/Syk/PI3K pathway and the infiltrated platelets promote the apoptosis of HCC cells. These findings confirm that platelets are important factors in tumor regulation.

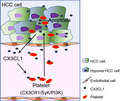

**Abbreviations**
CM: conditioned medium
CX3CL1: C‐X3‐C chemokine ligand 1
HCC: hepatocellular carcinoma
NT: nontumorous

## 1 Introduction

Hepatocellular carcinoma (HCC) is the second leading cause of cancer‐related death worldwide [1]. However, surgical treatment has a high risk of recurrence and metastasis [2]. Drug therapy can only prolong the survival time by few months [3]. Despite its promising therapeutic potential, cancer immunotherapy is also constantly encountering new challenges [4]. It is imperative to understand the pathogenesis of HCC and identify new treatment strategies.

Platelets have essential elements for migration, such as the expression of adhesion proteins and chemokine receptors, the release of matrix metalloproteinases, and the cytoskeleton and enzyme mechanisms necessary for cell movement, despite their lack of nucleus and limited protein synthesis [5]. Formyl peptides, cleavage products of bacteria, induced platelet migration at a velocity of 13.07 ± 1.10 μm·min^−1^ [6]. Lowenhaupt reported that the physiological substrate collagen induced platelets to migrate at a rate of 6 mm (3000 times of platelet diameter) per 15 min [7]. However, the structural features of collagen required for platelet aggregation are not necessary for collagen‐induced platelet chemotaxis, which does not require direct contact with platelets [8]. This suggests a fundamental difference between platelet migration and activation. In 2012, an article published in the *New England Journal of Medicine* reported that in addition to blood vessels, a large number of platelets appeared in the ovarian cancer tumor microenvironment [9]. However, the mechanism by which platelets exist outside the vessel and the role of migrating platelets in tumor are still unclear.

The tumor microenvironment is considered to be the inflammatory microenvironment with leukocytes. C‐X3‐C chemokine ligand 1 (CX3CL1) belongs to the CX3C chemokine/receptor family and is expressed in a variety of tumors [10]. CX3CL1 induces migration of immune cells by interacting with its receptor CX3CR1 [11]. CX3CR1 is also expressed in platelets [12], but whether CX3CL1 can induce platelet migration remains unknown.

Here, we found platelets migrated from blood vessels to HCC tissues, and CX3CL1 secreted by HCC cells directly induced platelet migration *in vitro* and *in vivo*, while CX3CR1 inhibition significantly blocked platelet recruitment. We found that migrating platelets promoted the apoptosis of HCC cells. Our current study reveals the molecular basis of platelet recruitment in HCC and provides new insights into the role of platelets in cancer.

## 2 Methods

### 2.1 Patient samples

Human HCC and their paired nontumorous (NT) liver samples were collected at Tongji Hospital affiliated to Huazhong University of Science and Technology, Wuhan, China. The tissue samples of 21 patients with HCC undergoing radical resection were confirmed pathologically as hepatocellular carcinoma. The patients were 53.2 ± 5.4 years old, with a median age of 53 years, 18 males, and 3 females. None had received chemotherapy or radiotherapy before surgery. The experiments were undertaken with the understanding and written consent of each subject, and the study methodologies were strictly in accordance with Helsinki declaration for the Use of Human Subjects and were approved by the Ethics Committee at Tongji Medical College, Huazhong University of Science and Technology (Wuhan, China; Approval No. IORG No: IORG0003571).

### 2.2 Cell culture and siRNA transfections

Hep3B, HepG2, SMMC.7721, HUVEC, and HL‐7702 were purchased from the Chinese Center for Type Culture Collection (CCTCC, Wuhan, China) and cultured in DMEM (HyClone, Logan, UT, US) supplemented with 10% FBS (Gibco, Grand Island, NY, USA). For hypoxia experiments, cells were incubated in 1% O_2_ and 5% CO_2_ at 37 °C. To generate conditioned medium, HCC cells were exposed to 20% or 1% O_2_ for 12 h, and cell culture supernatants were centrifuged at 1610 ***g*** for 15 min to remove cell sedimentation. The sequence of small interfering RNAs (siRNAs) targeting CX3CL1 was designed as TACCAACAAACAGCAAGTGGG and that of the nontarget control was designed as TTCTCCGAACGTGTCACGT. Vectors were stably transfected into HCC cell lines by lentiviral infection (LV‐Egf‐RNAi, 55844‐1), and media were changed after 24 h. The efficiency of siRNAs was measured by western blot.

### 2.3 Platelet preparation

Human venous blood was placed in a 10‐mL centrifuge tube. Each tube was 4 mL. 4 mL 2× Tyrode buffer was added to each tube and centrifuged at 160 ***g*** for 14 min. After finishing, the upper liquid (be careful not to suck out the white blood cells and red blood cells) was taken, PGE1 was added, and carefully blown and mixed with a pasteurized straw. After centrifugation at 500 ***g*** for 9 min, the white clumps at the bottom of the tube were platelets. The supernatant was carefully discarded, an appropriate amount of 1× Tyrode buffer added, and PGE1 and EDTA were added (final concentration: 1 mm), and then gently blown with a pasteurized pipette to resuspend platelets and centrifuged at 400 ***g*** for 9 min. Supernatant was carefully discarded and 1× Tyrode buffer and resuspended platelets were added. The number of platelets was measured by an automatic hematocyte analyzer and adjusted by 1× Tyrode buffer. The extraction process was carried out at room temperature.

### 2.4 Platelet migration analysis

For transmigration experiments, 12‐well Transwell inserts (Corning, New York, NY, USA) with 0.4‐μm membrane pores were used. The lower chamber contained cancer cell supernatant or human recombinant CX3CL1 (R&D Systems, Shanghai, China). Platelets or inhibitors pretreated platelets (5 × 10^5^/100 μL) were carefully transferred into the upper chamber and allowed to migrate for 8 h in a stable humidified atmosphere (5% CO_2_, pH 7.4, 37 °C). The platelets in the lower compartment were stained with glycoprotein αIIb (CD41a, BD, Franklin Lake, NJ, USA) antibody and counted by flow cytometry (BD). AZD8797 (5 μg·mL^−1^, CX3CR1 inhibitor), cytochalasin B (2 μg·mL^−1^, actin polymerization inhibitor), piceatannol (25 μm, Syk inhibitor), and LY294002 (40 μm, PI3K inhibitor) were purchased from MCE (Monmouth Junction, NJ, USA). The viability of inhibitor‐treated platelets and migrating platelets was confirmed by CCK8 assay.

### 2.5 Mitochondrial membrane potential and apoptosis detection

HCC‐siControl (siCN) or HCC‐siCX3CL1 cells (5 × 10^4^/well) were seeded in 6‐well plates and cultured overnight. Platelets (8 × 10^6^/500 μL) were added to the upper chamber (6‐well Boyden chamber with 0.4 μm pore size) and cocultured with HCC cells for 24 h, and the mitochondrial membrane potential and apoptosis of HCC cells were analyzed.

For collecting migrating platelets, platelets (8 × 10^6^/500 μL) were added to the upper chamber (6‐well Boyden chamber with 0.4 μm pore size), and CX3CL1 (Dispersed in DMEM, 10 ng·mL^−1^) was placed in the lower chamber. After 8 h, the platelet suspension of the lower chamber was centrifuged at 180 ***g*** for 3 min, followed by the removal of the supernatant, and the migrating platelets were resuspended in DMEM.

The mitochondrial membrane potential and apoptosis of HCC cells were analyzed by flow cytometry. After 24 h of coculture, the supernatant of the cell culture was collected into a 15‐mL centrifuge tube and gently washed with PBS twice. Trypsin digested cells for 3 min, DMEM culture medium terminated the digestion, and the cells were collected to the centrifuge tube containing the cell culture supernatant, centrifuged at 1200 r.p.m. for 5 min, and the supernatant was removed and washed in cold PBS. For mitochondrial membrane potential detection, preprepared JC‐1 working solution (1 mL/well) was added to the cell suspension and mixed gently. The cells were incubated at 37 °C for 20 min, centrifuged at 256 ***g*** for 5 min at 4 °C, and the supernatant was discarded. The cells were resuspended with 1× JC‐1 buffer and analyzed by flow cytometry. For apoptosis detection, the washed cells were recentrifuged, the supernatant was discarded, and cells were resuspended in 1× Annexin‐binding buffer. Five microlitre Alexa Fluor^®^ 488 Annexin V and 1 μL 100 μg·mL^−1^ PI working solutions were added to each 100 μL of cell suspension, and the cells were incubated at room temperature for 15 min. Four hundred microlitre 1× Annexin‐binding buffer was added, mixed gently, and analyzed by flow cytometry.

### 2.6 Immunohistochemistry

Formalin‐fixed, paraffin‐embedded tissue sections (5 μm) were stained with specific antibodies against CD41 (Santa Cruz, Dallas, TX, USA) (1 : 400), CD31 (1 : 500) (ProteinTech, Rosemont, IL, USA), CA9 (1 : 200) (Affinity, Cincinnati, OH, USA), or CX3CL1 (1 : 200) (Affinity). Images were captured using a fluorescence microscopy (Olympus BX51, Shinjuku‐ku, Tokyo, Japan).

### 2.7 Calcium mobilization assay

Platelets were suspended at 5 × 10^8^ mL^−1^ in 1× Tyrode buffer and pre‐incubated with AZD8797 or DMSO for 15 min and then incubated with 1 mm Fluo3‐AM (Dojindo Laboratories, Kyushu island, Japan) at room temperature for 40 min in the dark. After washing, platelets were resuspended at 5 × 10^6^·mL^−1^. Fluorescence intensity was measured for 30 s at 480 nm, and then, calcium chloride (final concentration 1 mm) and CX3CL1 (10 ng·mL^−1^) were added to incubate with platelets. They were detected immediately for 180 s after mixing gently. Results were normalized to initial fluorescence intensity.

### 2.8 Animal studies

Male BALB/c (nu/nu) mice aged 4–6 weeks were purchased from Beijing Huafukang Biotechnology Co., Ltd (Beijing, China). Mice were housed under specific pathogen‐free (SPF) conditions and cared according to the institutional guidelines for animal care. For orthotopic implantation, mice were deprived of food for 12 h and water for 4 h before surgery. The anesthesia was performed by intraperitoneal injection of 10% chloral hydrate, 0.1 mL/10 g. After disinfection with iodophor, the mice were fixed supine and a vertical opening (a length of 1.5 cm) was cut 1 cm below the xiphoid process. Skin, peritoneum, and muscle layers were cut layer by layer. Sterile gauze wetted with normal saline was spread under the wound, and ribs were gently pressed to extrude the left lobe of liver. 3 × 10^6^ (100 μL) SMMC.7721‐siCN or SMMC.7721‐siCX3CL1 cells were injected along the long axis of the liver lobe and in the direction of 20° with the liver plane. The injection was carried out slowly for 1 cm. After completion, the needle was quickly pulled out and the needle eye was gently pressed with a sterile cotton swab for 2 min. When there is no active bleeding, the leaking cells were gently wiped off with a cotton swab, the left lobe of the liver was gently returned to the abdominal cavity, gentamycin powder was sprinkled into the abdominal cavity, and abdomen was closed layer by layer. Postoperative water deprivation was for 3 h. Then, drink water and eat normally. Twenty‐eight days later, livers were harvested for analysis. All of the animal studies met the National Institutes of Health guidelines and were approved by the Committee on the Ethics of Animal Experiments of the Tongji Medical College, HUST [(2019) IACUC Number: 2330].

### 2.9 Western blot

For western blot, whole‐cell lysates of HCC cells were extracted and separated by 10% SDS/PAGE. The proteins were probed with antibodies against CX3CL1 (1 : 1000; ProteinTech, Wuhan, China), CX3CR1 (1 : 1000; ProteinTech), p‐Syk (Tyr525/526) (1 : 1000; ABclonal, Wuhan, China), Syk (1 : 1000; Cell Signaling Technology, Danvers, MA, USA), p‐Akt (Ser473) (1 : 1000; Cell Signaling Technology), or Akt (1 : 1000; Cell Signaling Technology). The protein bands were visualized using DNR Bio‐imaging Systems according to the manufacturer's instructions and quantified by IMAGE J (National Institutes of Health, Bethesda, MD, USA).

### 2.10 Statistics

Statistical analysis was performed using graphpad prism 7 (GraphPad Software, Inc., San Diego, CA, USA). Differences between groups were evaluated using 2‐tailed Student's *t*‐test or one‐way ANOVA, adjusting for multiple comparisons. Results are presented as the mean ± SEM. For all statistical analyses, *P* < 0.05 was considered statistically significant.

## 3 Results

### 3.1 Platelet infiltration into HCC tissue

**Fig. 1:**
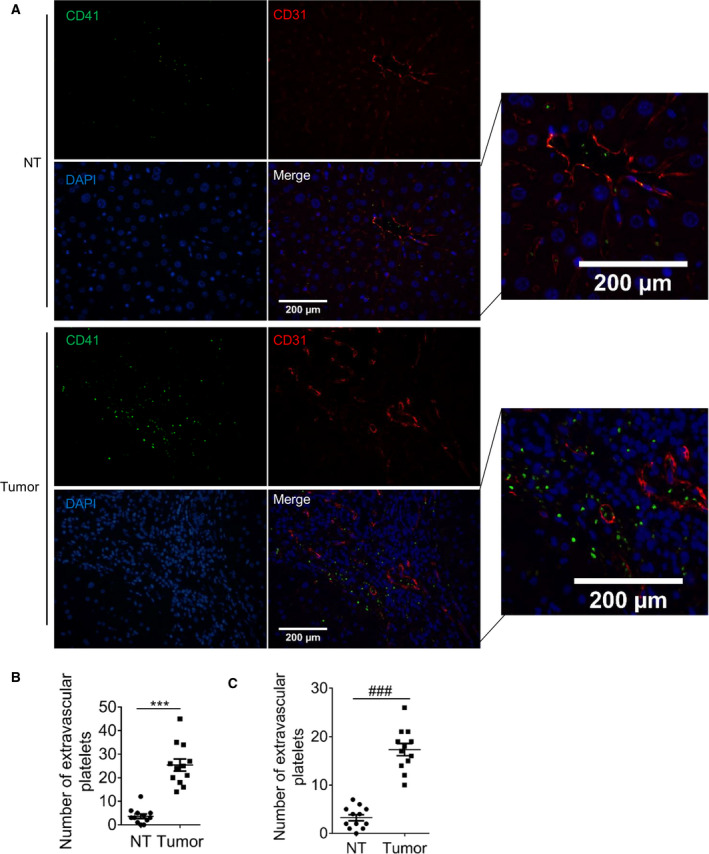
Platelets are present outside the blood vessels in HCC tissues. Human HCC tissues and adjacent nontumor (NT) liver tissues were stained with platelet‐specific marker CD41 (green), vascular endothelial molecule CD31 (red), and DAPI (blue). (A) The representative images (*n* = 4, random three fields per case). Bar, 200 μm. (B) The number of platelets. (C) Platelet number in C57 orthotopic tumor (*n* = 4, random three fields per case). Values are shown as dots and the mean ± SEM, ***P < 0.001, ^###^ *P* < 0.001 (paired *t*‐test, 2‐tailed).

Platelets are thought to have the ability to migrate and penetrate lung and subcutaneous tissue. We observed the presence of platelets (indicated by CD41, a platelet‐specific marker) outside the blood vessels (indicated by CD31) in human HCC tissues, but not in adjacent nontumor (NT) liver tissue (Fig. 1A,B). The diameter of CD41‐positive particles was 3–10 μm in human HCC tissues, slightly larger than normal human platelets (2–5 μm) [13]. In addition, platelet infiltration *in situ* tumor tissues of HCC in C57 mice was also observed (Fig. 1C, Fig. S1).

### 3.2 CX3CL1 induces platelet migration

**Fig. 2:**
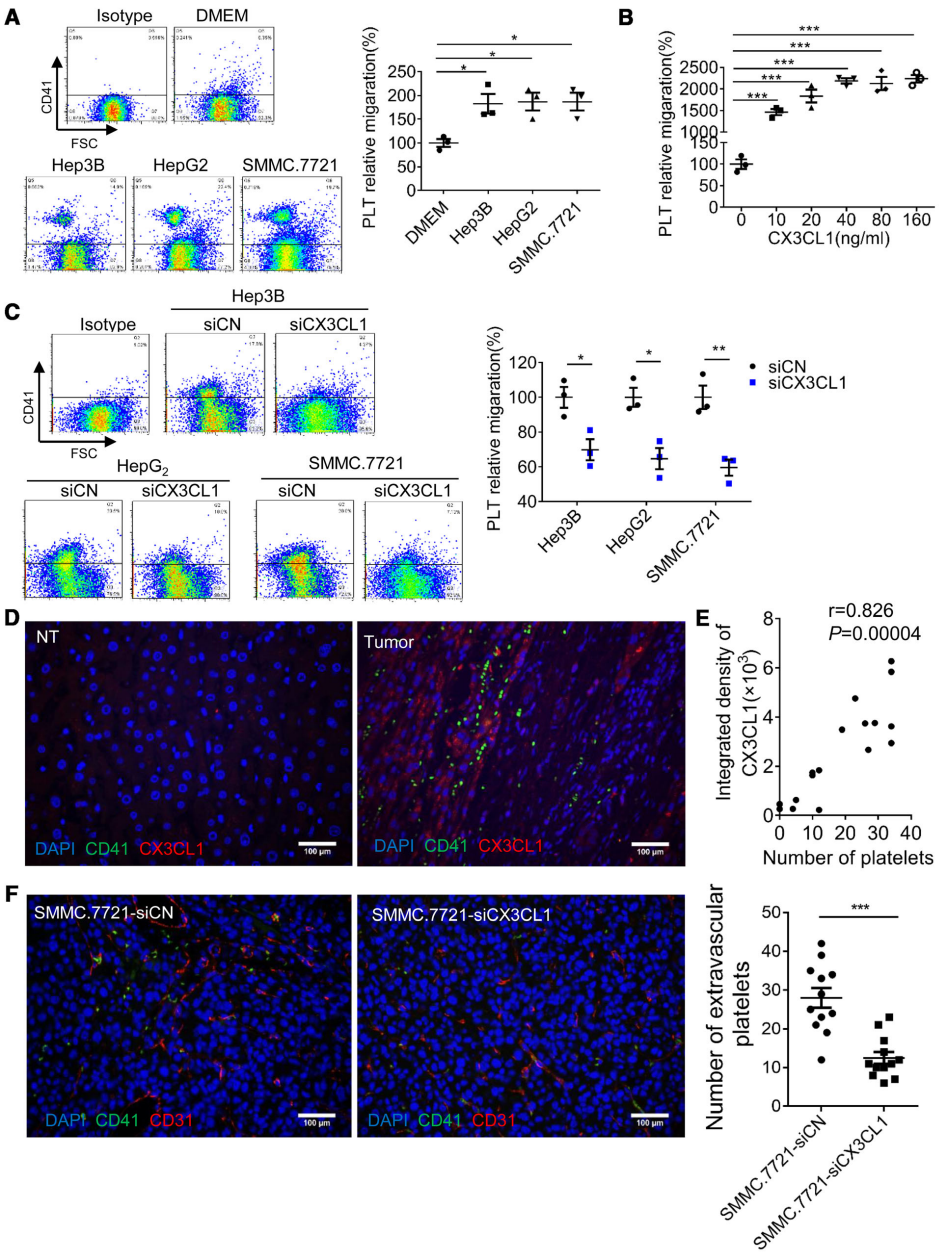
CX3CL1 induces platelet migration. Platelet migration induced by HCC cell CM (A, *n* = 3, means ± SEM, 1‐way ANOVA, *P < 0.05, **P < 0.01), human CX3CL1 recombinant protein (B, *n* = 3, means ± SEM, 1‐way ANOVA, ***P < 0.001), and HCC cell CM (C, *n* = 3, means ± SEM, unpaired *t*‐test, 2‐tailed, *P < 0.05, **P < 0.01). (D) Platelet infiltration and CX3CL1 expression in human HCC tissues and NT liver tissues (*n* = 7, random 2–3 fields per case). Bar, 100 μm. (E) Correlation analysis of platelet number and CX3CL1 expression (400× field of view, Spearman's rank correlation). (F) An orthotopic model of HCC in nude mice was constructed by SMMC.7721‐siCN and SMMC.7721‐siCX3CL1 cells, and platelet infiltration was analyzed in the 400× field of view (*n* = 4, random three fields per case, unpaired *t*‐test, 2‐tailed, mean ± SEM, ***P < 0.001). Bar, 100 μm. PLT, platelet.

Sinusoidal endothelial cells have many fenestrae, most of which are between 50 and 300 nm in diameter and a few between 500 and 2000 nm in diameter [14, 15]. In addition, there is no regular basal lamina under the endothelial cell. To analyze platelet migration, Transwell inserts with 0.4‐μm membrane pores were used and endothelial cells were not covered on the membrane. Flow cytometry confirmed the size of CD41‐positive particles was consistent with that of platelets (Fig. S2). CCK8 assay confirmed the viability of migrating platelets (Fig. S3). Compared with DMEM, conditioned medium (CM) generated by Hep3B, HepG2, and SMMC.7721 cells induced platelet migration (Fig. 2A). It has been reported that CX3CL1 is a regulator of NK cell and T‐cell recruitment [16, 17]. We found CX3CL1 recombinant protein also induced platelet migration and the number of migrating platelets was concentration‐dependent (Fig. 2B). When CX3CL1 concentration reached 40 ng·mL^−1^, platelet migration did not increase significantly and reached a stable level. We downregulated CX3CL1 expression in HCC cells (Fig. S4A) and reduced platelet migration (Fig. 2C). In human HCC tissues, we found there was more platelet infiltration in the CX3CL1 expression region, while almost no platelet infiltration in NT tissues (Fig. 2D). Platelet count was positively correlated with CX3CL1 expression (Fig. 2E). To confirm the role of CX3CL1 in platelet recruitment, 1 × 10^6^ SMMC.7721‐siCN and SMMC.7721‐siCX3CL1 cells were orthotopically implanted into the livers of BALB/c nude mice. Results showed that compared with siCN group, the platelet infiltration in siCX3CL1 group was significantly reduced (Fig. 2F). In summary, HCC cell‐derived CX3CL1 induces platelet migration.

### 3.3 CX3CR1/Syk/PI3K is essential for CX3CL1‐induced migration

**Fig. 3:**
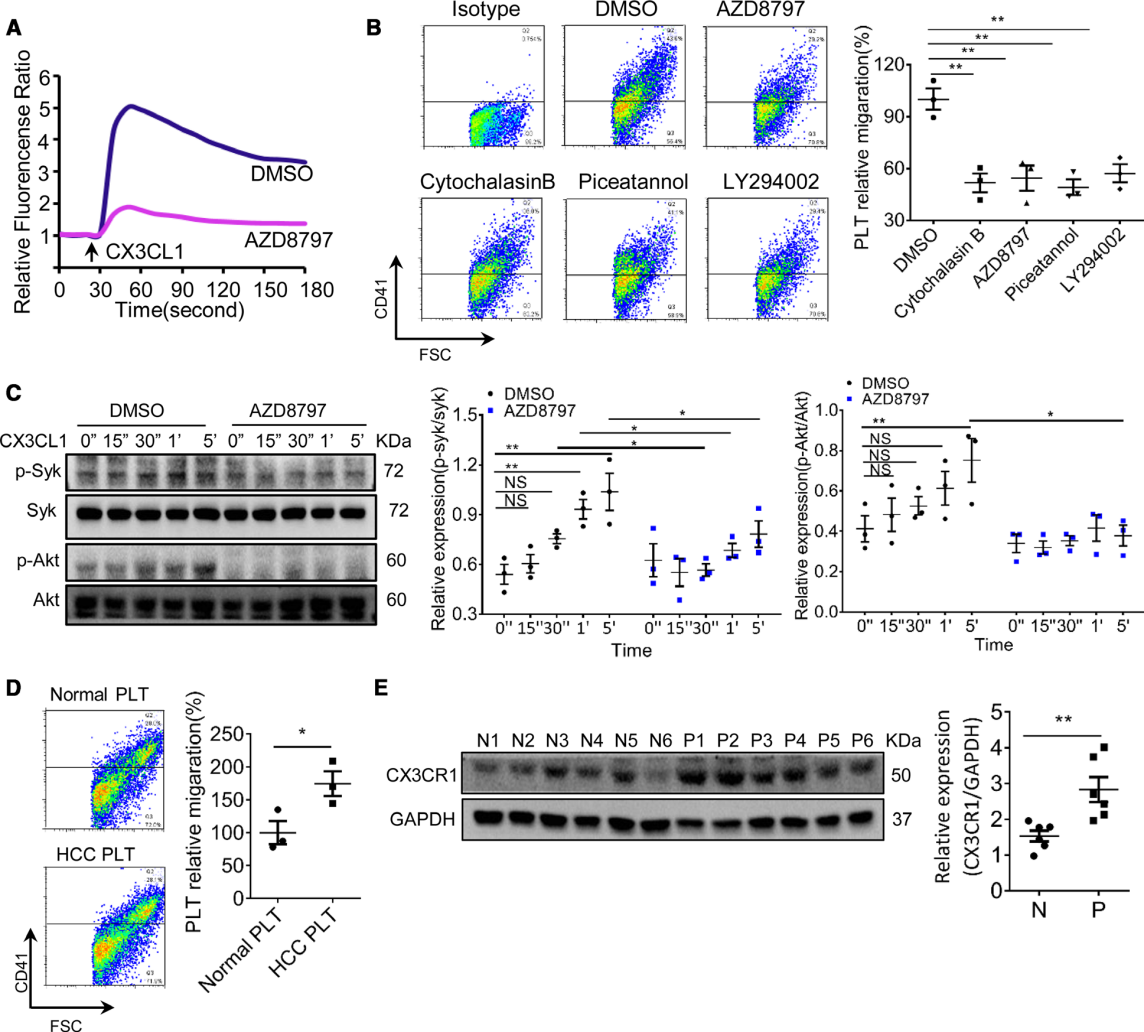
CX3CR1/Syk/PI3K is essential for CX3CL1‐induced platelet migration. (A) Platelet intracellular calcium flux was measured by Fluo3‐AM, and the fitting curves were shown (*n* = 3). (B) The analysis of platelet migration (*n* = 3, 1‐way ANOVA, mean ± SEM, **P < 0.01). (C) The expression of p‐Syk, Syk, p‐Akt, and Akt in platelet, and all the blots were derived from the same samples (*n* = 3, unpaired *t*‐test, 2‐tailed, mean ± SEM, *P < 0.05, **P < 0.01, NS, no significant difference). (D) HCC patients' platelet migration (*n* = 3, unpaired *t*‐test, 2‐tailed, mean ± SEM, *P < 0.05). (E) The expression of CX3CR1 in normal (N, *n* = 6) and HCC patients' platelets (P, *n* = 6), and the blots were derived from the same gel (unpaired *t*‐test, 2‐tailed, mean ± SEM, **P < 0.01).

Intracellular calcium mobilization is essential for cytoskeletal rearrangement and cell movement. We found CX3CL1 at 10 ng·mL^−1^ could induce calcium signaling in platelets, whereas platelets incubated with CX3CR1 antagonist AZD8797 were less sensitive to CX3CL1, and there was no significant change in intracellular calcium flux (Fig. 3A). Inhibition of CX3CR1 or inhibition cytoskeletal rearrangement significantly reduced platelet migration (Fig. 3B). These results confirm that CX3CR1 is involved in CX3CL1‐induced platelet migration. In neutrophils, Syk has been shown to be necessary for G‐protein‐coupled receptor‐mediated chemotaxis [18] and participates in CX3CL1‐mediated macrophage migration via CX3CR1/Syk/Cdc42/WASP and CX3CR1/Syk/Rac1/WAVE2 [19]. PI3K plays an important role in chemotaxis of T cells and B cells, and the activation of PI3K is closely related to Syk [20]. Here, we analyzed the role of Syk and PI3K in CX3CL1‐induced platelet migration. Blocking Syk or PI3K, respectively, significantly reduced platelet migration (Fig. 3B). This suggests that Syk/PI3K plays a key role in CX3CL1‐induced platelet migration. We further found CX3CL1 recombinant protein induces phosphorylation of Syk and Akt in a time‐dependent manner, and blocking CX3CR1 significantly attenuated this effect (Fig. 3C). These results confirm Syk/PI3K is downstream of CX3CR1. We found that HCC patients' platelets migrated faster than normal platelets under the same condition (Fig. 3D). And CX3CR1 expression was higher in HCC platelets (Fig. 3E). Increased platelet migration may be related to the higher expression of CX3CR1.

### 3.4 Hypoxia promotes platelet recruitment by upregulating CX3CL1 expression

**Fig. 4:**
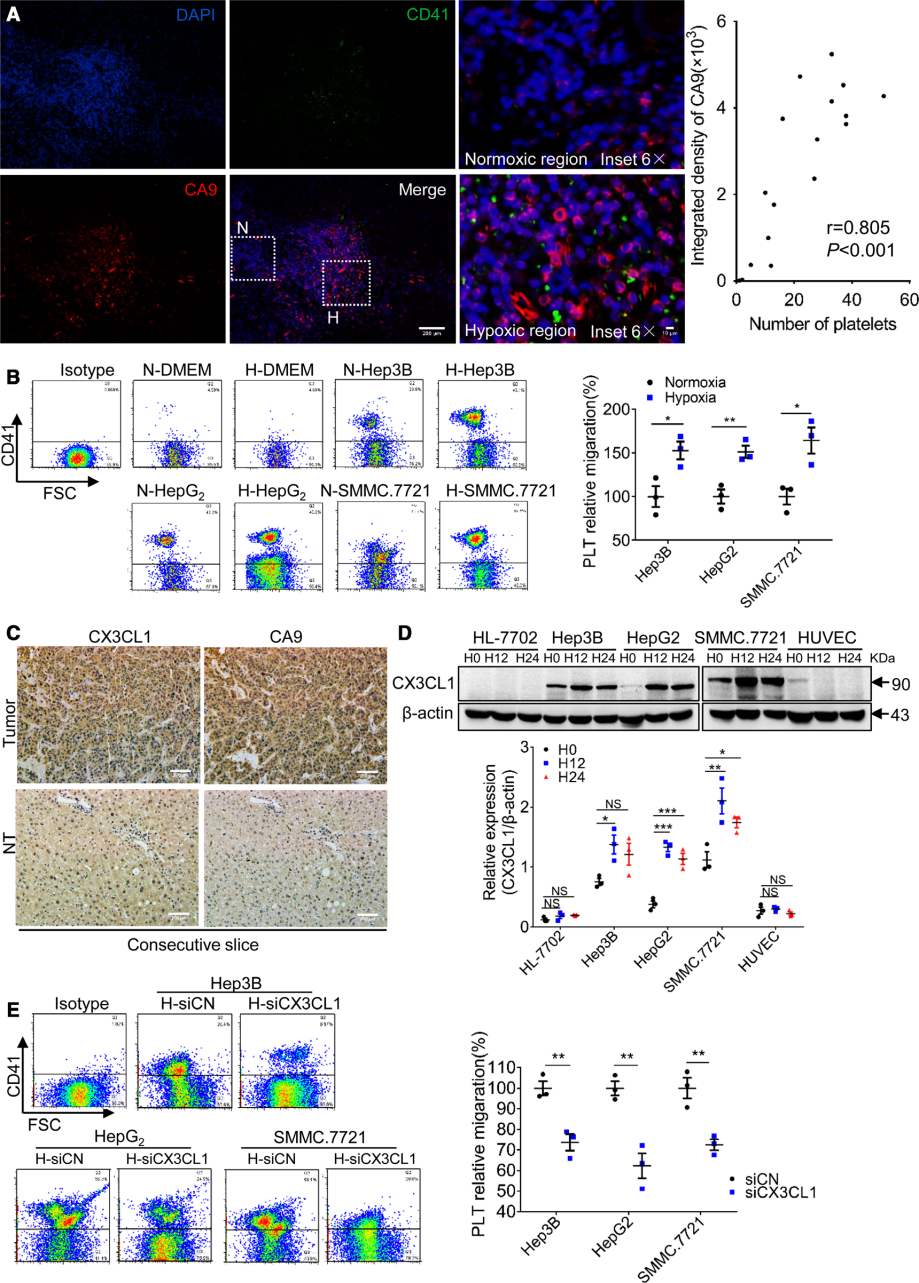
Hypoxia promotes platelet recruitment by upregulating CX3CL1 expression. (A) Representative fluorescence images of platelets (CD41) and hypoxia (CA9) on section of human HCC and the correlation analysis of platelet number versus CA9‐integrated density (*n* = 5, random 3–4 fields per case; Spearman's rank correlation). Bar, 200 μm. The dotted lines enclose the hypoxic (H) and normoxic (N) areas in the HCC tissue, respectively. Bar, 10 μm. (B) Platelet migration assay (*n* = 3, unpaired *t*‐test, 2‐tailed, mean ± SEM, *P < 0.05, **P < 0.01). (C) Expression of CX3CL1 and CA9 in human HCC and NT liver tissues. Representative images of two consecutive slices of the same sample embedded in paraffin were taken, and each was stained with CX3CL1 and CA9 (*n* = 3). Bar, 200 μm. (D) CX3CL1 protein expression, and the blots were derived from the same gel (*n* = 3, 1‐way ANOVA or unpaired *t*‐test, 2‐tailed, mean ± SEM, *P < 0.05, **P < 0.01, ***P < 0.001, NS, no significant difference). (E) Platelet migration assay (*n* = 3, unpaired *t*‐test, 2‐tailed, mean ± SEM, **P < 0.01).

In human liver cancer tissues, there was strong CA9 signal (hypoxia marker) in the area of platelet infiltration, but less in the area with weak CA9 signal. Platelet count was positively correlated with CA9 expression (Fig. 4A). This suggests that platelet infiltration may be related to hypoxia. The results showed that hypoxic CM had a greater effect on platelet recruitment (Fig. 4B). The expression of CX3CL1 was reported to be regulated by hypoxia [21]. And we found that CX3CL1 had a strong signal in the hypoxic areas of human HCC tissues, while the signal was weak in the nonhypoxic NT tissues (Fig. 4C). At 12 h of hypoxia, the expression of CX3CL1 in HCC cells reached a peak, while CX3CL1 was not expressed in HL‐7702 and HUVEC cells under normal and hypoxic conditions (Fig. 4D). To further demonstrate the mechanistic link between hypoxia and platelet recruitment, we placed the CM generated by hypoxic HCC‐siCN and HCC‐siCX3CL1 cells in the lower chamber to analyze platelet migration. After CX3CL1 was knocked out, the migration induced by hypoxic CM was significantly reduced (Fig. S4B, Fig. 4E). These confirm that hypoxia‐induced platelet infiltration is closely related to high expression of CX3CL1 in HCC cells.

### 3.5 Migrating platelets promote apoptosis of HCC

**Fig. 5:**
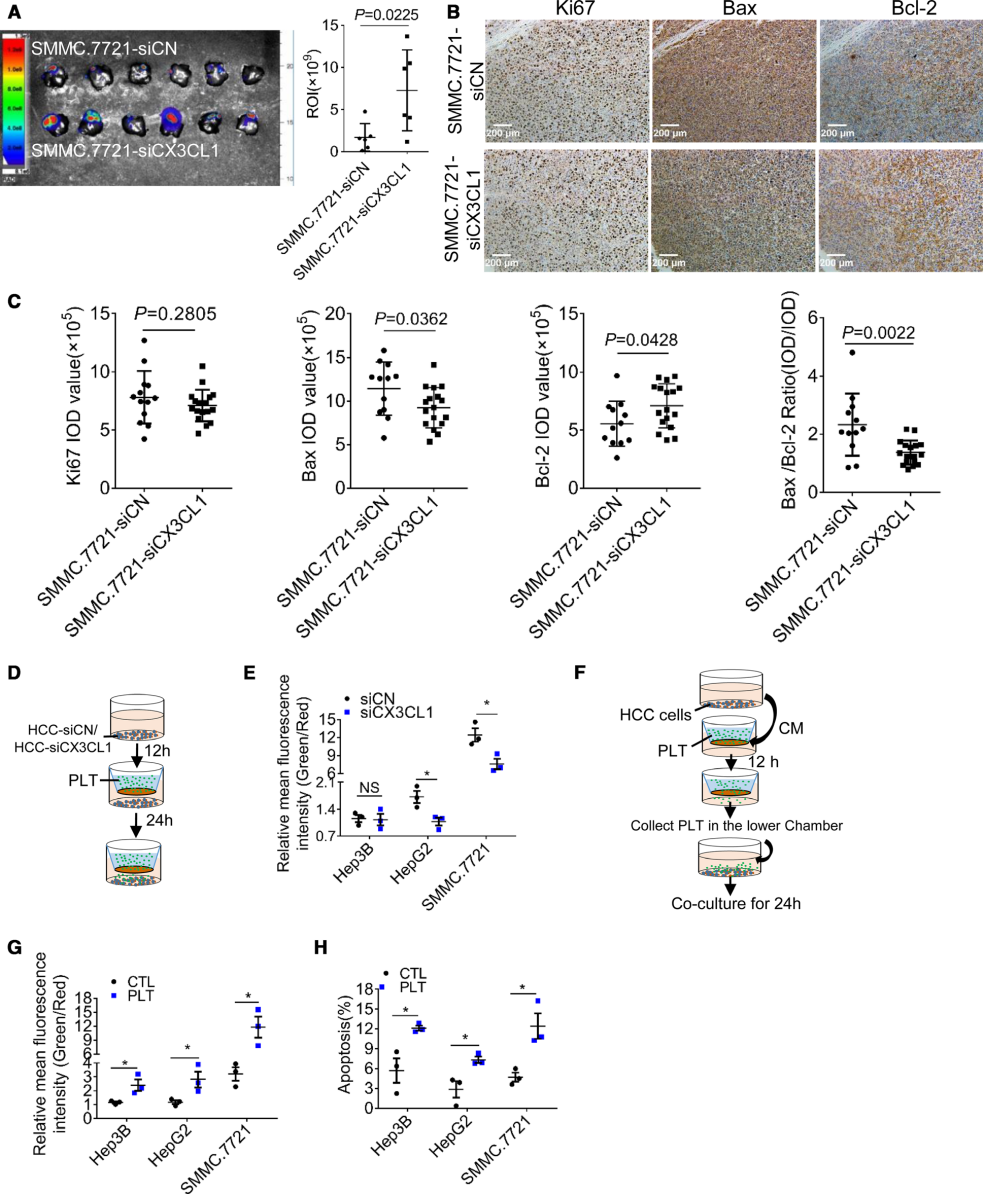
Migrating platelets promote the apoptosis of HCC cells. (A) The dissected livers form orthotopic model of HCC in nude mice (*n* = 6 for each group), and the fluorescence intensity of tumors (regions of interest, ROI) was analyzed. (B) Tumors were stained with proliferation marker Ki67 and apoptosis marker Bax, and Bcl‐2 and the representative images were shown (*n* = 4, random three fields per case). Bar, 200 μm. (C) The integrated option density (IOD) of Ki67, Bax, and Bcl‐2 (*n* = 4, random three fields per case) and the ratio of Bax/Bcl‐2 were analyzed (unpaired *t*‐test, 2‐tailed, means ± SEM). (D) Experiment design, and si‐CX3CL1 or siCN HCC cells were placed in the lower chamber of Transwell. After 12 h, platelets were added to the upper chamber. The apoptosis of hepatocellular carcinoma cells was analyzed 24 h later. (E) Mitochondrial membrane potential analyzed by JC‐1. (F) Experiment design. HCC cell supernatant was placed in the lower chamber of Transwell, and platelets were added to the upper chamber. After 12 h, the migrated platelets were collected and cocultured with HCC cells for 24 h. (G) The mitochondrial membrane potential of HCC cells. (H) The apoptosis of HCC cells. Results of E, G, and H are expressed as means ± SEM (*n* = 3, unpaired *t*‐test, 2‐tailed). NS, no significant difference, *P < 0.05.

Compared with siCN cells, platelet infiltration in SMMC.7721‐siCX3CL1 cell‐derived tumors was significantly reduced. However, the tumor size in SMMC.7721‐siCX3CL1 group was significantly larger than that of SMMC.7721‐siCN group (Fig. 5A). There was no significant difference in the expression of proliferation marker Ki67 between the two groups. The expression of Bax and Bcl‐2 in the SMMC.7721‐siCX3CL1 group was lower and higher than those in siCN group. In SMMC.7721‐siCX3CL1 group, the ratio of Bax/Bcl‐2 was lower than that of SMMC.7721‐siCN group (Fig. 5B,C). These results indicate that CX3CL1 negatively regulates the growth of hepatocellular carcinoma. We found no significant difference in cell viability between SMMC.7721‐siCN and SMMC.7721‐siCX3CL1 cells under normoxia and hypoxia conditions (Fig. S5). It has been reported that CX3CL1 can induce immune cell infiltration and participate in antitumor immunity [22]. Platelet infiltration is also regulated by CX3CL1, and the role of CX3CL1 has aroused our attention.

To investigate the effect of platelet infiltration on HCC cells, platelets were cocultured with HCC‐siCN or HCC‐siCX3CL1 cells through Transwell (Fig. 5D). The result showed that the mitochondrial membrane potential of HCC‐siCX3CL1 cells was lower than that of HCC‐siCN cells (Fig. 5E, Fig. S6A). This suggests that reducing the number of migrating platelets rescues the decrease in mitochondrial membrane potential of HCC cells. Next, platelets in the low chamber were collected and cocultured with HCC cells (Fig. 5F). We found platelets profoundly reduced mitochondrial membrane potential of HCC cells (Fig. 5G, Fig. S6B), increased the proportion of apoptosis (Fig. 5H, Fig. S6C), and increased the level of cleaved caspase 3 in HCC cells (Fig. S7), suggesting that migrating platelets induce apoptosis of HCC cells.

## 4 Discussion

In addition to regulating hemostasis and coagulation, platelets are also key regulators of tumor progression. The direct interaction between platelets and tumor cells is considered to be the contact between platelets and circulating tumor cells *in vivo*. Our study shows that platelets are present in HCC tissues, and CX3CL1 directly induces platelet migration, which can be significantly enhanced in the case of hypoxia. In addition, migrating platelets promote apoptosis of HCC cells.

In human liver, hepatic sinusoidal endothelial cells possess thin and flat cytoplasm and the thinnest parts of cell cytoplasm (0.1 μm) frequently possessed numerous fenestrations of various diameters [15]. Normally, the sinusoidal endothelium will inhibit the entry of platelets into the Disse spaces through its filtering function. Here, we found platelets infiltrated HCC tissue. Platelet infiltration also exists in LPS‐induced liver injury models. 10–20% of the platelets were trapped in the sinusoidal and Disse's spaces at 4.5 h after LPS injection (0.1 mg·kg^−1^) [23]. Platelet has strong deformability and has been shown to be mobile. We suspect that platelets actively enter the subendothelial space. Sinusoidal endothelial cells have specialized cytoplasm much thinner than platelets and have clustered fenestrations, which provide the structural basis for platelet passage. In addition, the increase in fenestration size under hypoxia conditions may also be a contributing factor [24]. It has been reported that endothelial cells were altered in human HCC tissue [25]. The endothelial fenestrae disappeared, and the cells were arranged in multiple layers and connected with each other by desmosomes. Sinusoidal endothelial cells, which no longer exchange substances, act as a ‘wall’ protecting tumor cells from invasion and destruction by immune cells. Platelets have been reported to regulate endothelial cells and affect small vascular permeability [26], and platelet microparticles also mediate endothelial injury in early diabetic nephropathy [27]. Studies have found that platelets migrate through endothelial cells, and activation of endothelial cells promotes platelet migration [28]. We cannot rule out the possibility that the infiltration of platelets in the HCC tissue is the result of interaction between sinusoidal endothelial cells and platelets. We found HCC cell‐derived CX3CL1 induces platelet migration in hypoxia microenvironment. The HCC microenvironment is not simply anoxic and has many inflammatory factors. Inflammatory factors such as TNF‐α have been shown to upregulate CX3CL1 expression in endothelial cells [29]. Upregulation of CX3CL1 expression in endothelial cells may be the promoter of platelet recruitment, but not the driver of platelet entering the subendothelial space.

The high expression of CX3CL1 and CX3CR1 significantly reduces recurrences and improves prognosis in HCC [30]. CX3CL1 reduces primary tumor growth and spontaneous liver metastases [31]. Immune cells play an important regulatory role in tumor microenvironment. NK cells, T cells, and dendritic cells are involved in CX3CL1‐induced antitumor immunity [16, 32], and macrophages exhibit tumor‐promoting characteristics different from circulating macrophages [33, 34]. We found platelets recruited by CX3CL1 promoted the apoptosis of HCC cell. Studies have shown that platelets secrete growth factors that promote tumor development, and platelet secretions are the result of high activation and even aggregation. We found no significant platelet aggregation in HCC tissues. This suggests that the infiltrated platelets may not have the properties of aggregating platelets to release growth factors. This may be the cause of hepatocellular carcinoma cell apoptosis. The inhibitory effect of platelets on tumor has been reported. Human platelets are rich in Fas‐L, which can be expressed rapidly on the surface of platelets after activation. Fas‐L expression can induce apoptosis of Fas‐positive tumor cells [35, 36]. Besides, platelets are rich in microRNAs. Platelet‐derived miRNA‐24 induces mitochondrial dysfunction and apoptosis in colon cancer cells [37]. Whether the platelets infiltrating tumor tissues are different from circulating platelets and the mechanism of platelet recruitment inducing apoptosis of HCC cells remains to be further studied. Clarifying the relationship between platelet and HCC will provide a strong theoretical basis for the prevention and treatment of HCC.

## 5 Conclusions

Platelets are important factors in tumor regulation. The role of platelets in the HCC microenvironment deserves our attention. For the first time, we found that CX3CL1 induced platelet active infiltration into the HCC microenvironment through CX3CR1/Syk/PI3K, and the recruited platelets participated in the regulation of HCC cell apoptosis.

## Conflict of interest

The authors declare no conflict of interest.

## Author contributions

SM performed all the experiments. ML and YL collected clinical specimens, and DS and WS conducted animal experiments. YM and YZ carried out the statistical analysis. RM, BZ, and CF analyzed and interpreted the data. SM and Z‐YM wrote the manuscript. SM and Z‐YM conceived the ideas and designed the experiments. All authors have reviewed the manuscript. All authors read and approved the final manuscript.

## Supporting information


**Fig. S1.** Platelets are present outside the blood vessels in C57 orthotopic tumor tissues.

**Fig. S2.** Migrating platelets analyzed by Flow Cytometry.

**Fig. S3.** Platelet activity analysis.

**Fig. S4.** Analysis of CX3CL1 knockdown efficiency.

**Fig. S5.** Cell viability analysis of HCC cells by CCK8 assay.

**Fig. S6.** Migrating platelets promote the apoptosis of HCC cells.

**Fig. S7.** Recruited platelets increase the level of cleaved caspase 3 in HCC cells.

## Data Availability

All data generated or analyzed during this study are included in this published article and supplementary materials. The raw data are available from the corresponding author upon reasonable request.
