# Supplementary material for: Platelets are recruited to hepatocellular carcinoma tissues in a CX3CL1‐CX3CR1 dependent manner and induce tumour cell apoptosis
Source: Mol Oncol. 2020 Sep 2;14(10):2546–59. doi: 10.1002/1878-0261.12783 (PMC7530782; doi:10.1002/1878-0261.12783)
Supplement: Supplementary file 1 — Fig. S1. Platelets are present outside the blood vessels in C57 orthotopic tumor tissues. [file MOL2-14-2546-s001.pdf]

Supplementary figures

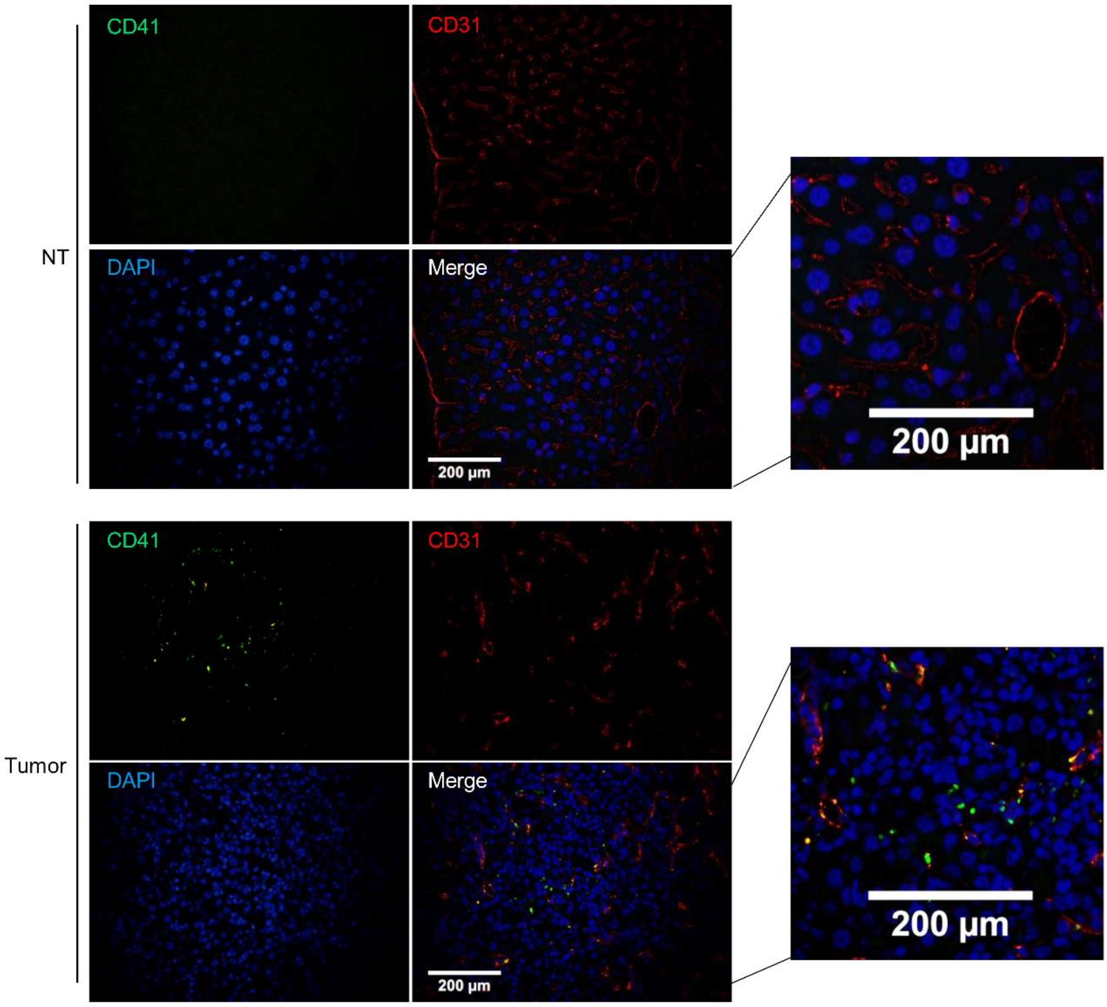

Supplementary Fig. 1. Platelets are present outside the blood vessels in C57 orthotopic tumor tissues. C57 mouse orthotopic tumor and their liver NT tissues were stained with platelet-specific marker CD41 (green), vascular endothelial molecule CD31 (red) and DAPI (blue), and the representative images were shown (n=4, random 3 fields per case). Bar, 200 µm.
