# Supplementary material for: Platelets are recruited to hepatocellular carcinoma tissues in a CX3CL1‐CX3CR1 dependent manner and induce tumour cell apoptosis
Source: Mol Oncol. 2020 Sep 2;14(10):2546–59. doi: 10.1002/1878-0261.12783 (PMC7530782; doi:10.1002/1878-0261.12783)
Supplement: Supplementary file 2 — Fig. S2. Migrating platelets analyzed by Flow Cytometry. [file MOL2-14-2546-s002.pdf]

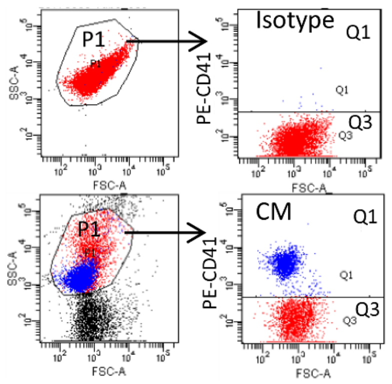


**Supplementary Fig. 2. Migrating platelets analyzed by Flow Cytometry.** A gate (left top panel) was drawn using freshly isolated platelets and the CD41-positive particles in this gate are considered to be platelets. Platelets incubated with PE-CD41 isotype antibody were used to distinguish CD41 negative and positive ones, and thus a dividing line was drawn (right top panel). Samples were collected at the same time using the same flow rate to ensure the same volume is collected and we convert the number of migrating platelets to relative percentage based on the control group (n=3).
