# Supplementary material for: Platelets are recruited to hepatocellular carcinoma tissues in a CX3CL1‐CX3CR1 dependent manner and induce tumour cell apoptosis
Source: Mol Oncol. 2020 Sep 2;14(10):2546–59. doi: 10.1002/1878-0261.12783 (PMC7530782; doi:10.1002/1878-0261.12783)
Supplement: Supplementary file 3 — Fig. S3. Platelet activity analysis. [file MOL2-14-2546-s003.pdf]

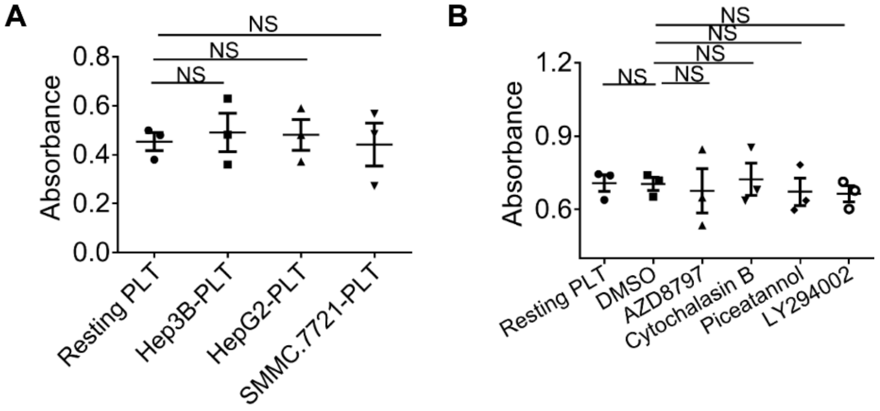

Supplementary Fig. 3. Platelet activity analysis. For activity analysis, migrating platelets or inhibitors treated platelets were collected and added to 96 wells and the activity of platelets was analyzed by CCK8 assay according to the manufacturers' protocols. (A) Migrating platelets showed similar absorbance as the resting platelets. This confirm that platelets were still alive after 8 hours of migration (n=3, mean ± SEM, 1-way ANOVA with Tukey's multiple comparisons test. NS, no significant difference.). Hep3B-PLT: migrating platelets induced by Hep3B CM. HepG2 and SMMC.7721 are the same. (B) Inhibitors did not affect platelet activity. CytochalasinB (2μg/ml), AZD8797 (10nM), Piceatannol (25μM), LY294002 (40μM) or DMSO (1.5μl/500μl) were incubated for 15 minutes (n=3, mean ± SEM, 1-way ANOVA with Tukey's multiple comparisons test. NS, no significant difference.).
