# Supplementary material for: Platelets are recruited to hepatocellular carcinoma tissues in a CX3CL1‐CX3CR1 dependent manner and induce tumour cell apoptosis
Source: Mol Oncol. 2020 Sep 2;14(10):2546–59. doi: 10.1002/1878-0261.12783 (PMC7530782; doi:10.1002/1878-0261.12783)
Supplement: Supplementary file 4 — Fig. S4. Analysis of CX3CL1 knockdown efficiency. [file MOL2-14-2546-s004.pdf]

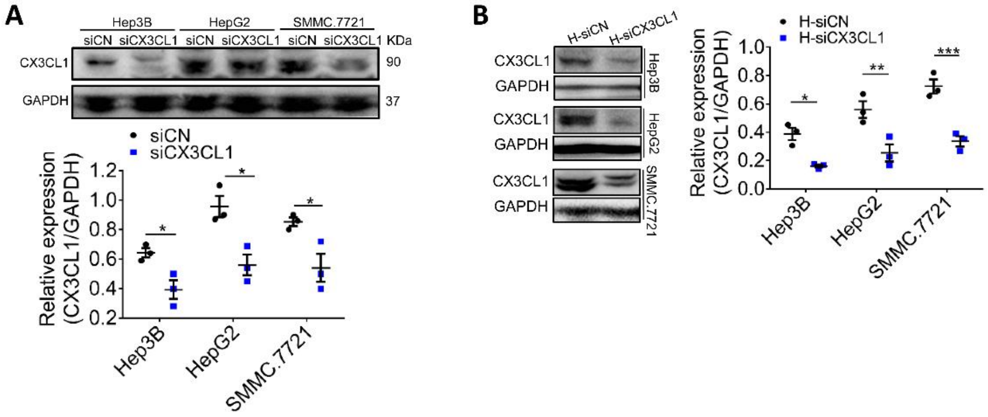

Supplementary Fig.4. Analysis of CX3CL1 knockdown efficiency. (A) Expression of CX3CL1 in HCC cells transfected with siCX3CL1 or siCN under normoxia (n=3, unpaired t test, 2-tailed, mean ± SEM, **P*<0.05). (B) HCC cells transfected with siCX3CL1 or siCN were cultured for 12 hours under hypoxia to detect CX3CL1 expression (n=3, unpaired t test, 2-tailed, mean ± SEM, **P*<0.05, ***P*<0.01, ****P*<0.001).
