# Supplementary material for: Platelets are recruited to hepatocellular carcinoma tissues in a CX3CL1‐CX3CR1 dependent manner and induce tumour cell apoptosis
Source: Mol Oncol. 2020 Sep 2;14(10):2546–59. doi: 10.1002/1878-0261.12783 (PMC7530782; doi:10.1002/1878-0261.12783)
Supplement: Supplementary file 5 — Fig. S5. Cell viability analysis of HCC cells by CCK8 assay. [file MOL2-14-2546-s005.pdf]

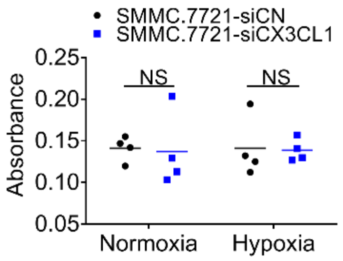

Supplementary Fig.5. Cell viability analysis of HCC cells by CCK8 assay. SMMC.7721-siCN and SMMC.7721-siCX3CL1 cells were seeded in 96-well plates (5000/well), and after 12 hours of starvation treatment, they were cultured for 24 hours under normoxia and hypoxia, respectively(n=4, unpaired t test, 2-tailed, mean ± SEM. NS, no significant difference.).
