# Supplementary material for: Platelets are recruited to hepatocellular carcinoma tissues in a CX3CL1‐CX3CR1 dependent manner and induce tumour cell apoptosis
Source: Mol Oncol. 2020 Sep 2;14(10):2546–59. doi: 10.1002/1878-0261.12783 (PMC7530782; doi:10.1002/1878-0261.12783)
Supplement: Supplementary file 6 — Fig. S6. Migrating platelets promote the apoptosis of HCC cells. [file MOL2-14-2546-s006.pdf]

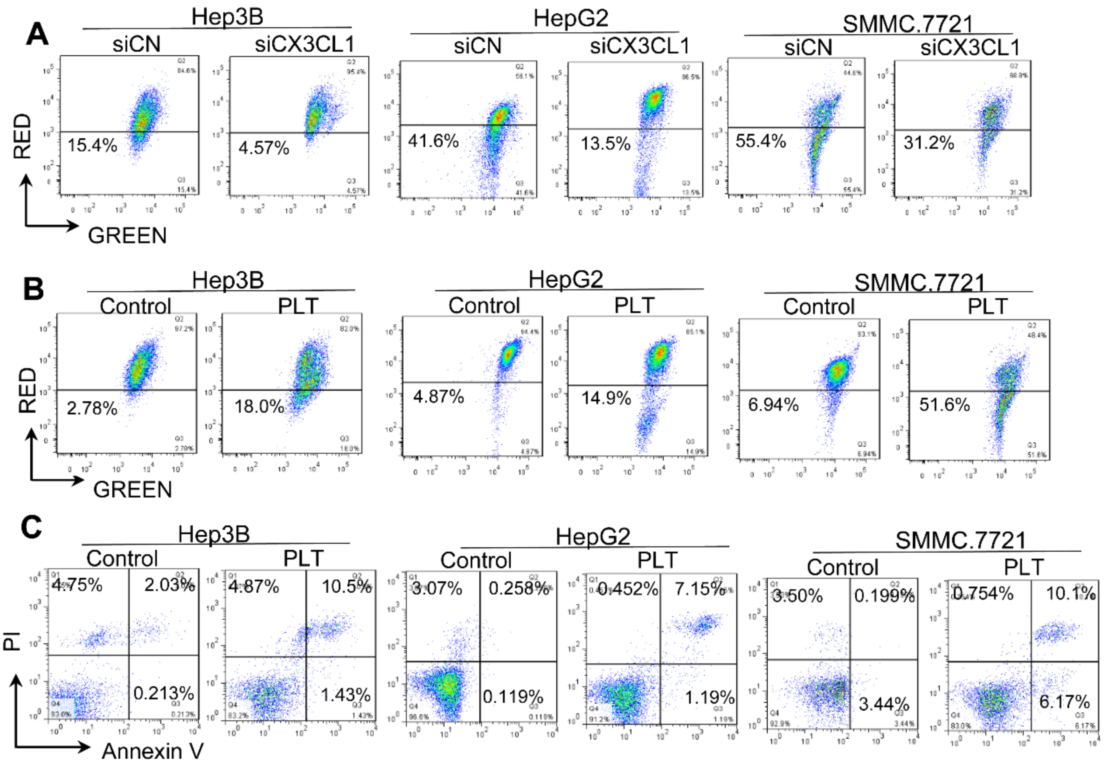

Supplementary Fig. 6. Migrating platelets promote the apoptosis of HCC cells. Mitochondrial membrane potential of HCC cells analyzed by JC-1 and representative images were shown (A and B, n=3, respectively.). (C) Analysis of the apoptosis of HCC cells by Flow Cytometry (n=3).
