# Supplementary material for: Platelets are recruited to hepatocellular carcinoma tissues in a CX3CL1‐CX3CR1 dependent manner and induce tumour cell apoptosis
Source: Mol Oncol. 2020 Sep 2;14(10):2546–59. doi: 10.1002/1878-0261.12783 (PMC7530782; doi:10.1002/1878-0261.12783)
Supplement: Supplementary file 7 — Fig. S7. Recruited platelets increase the level of cleaved caspase 3 in HCC cells. [file MOL2-14-2546-s007.pdf]

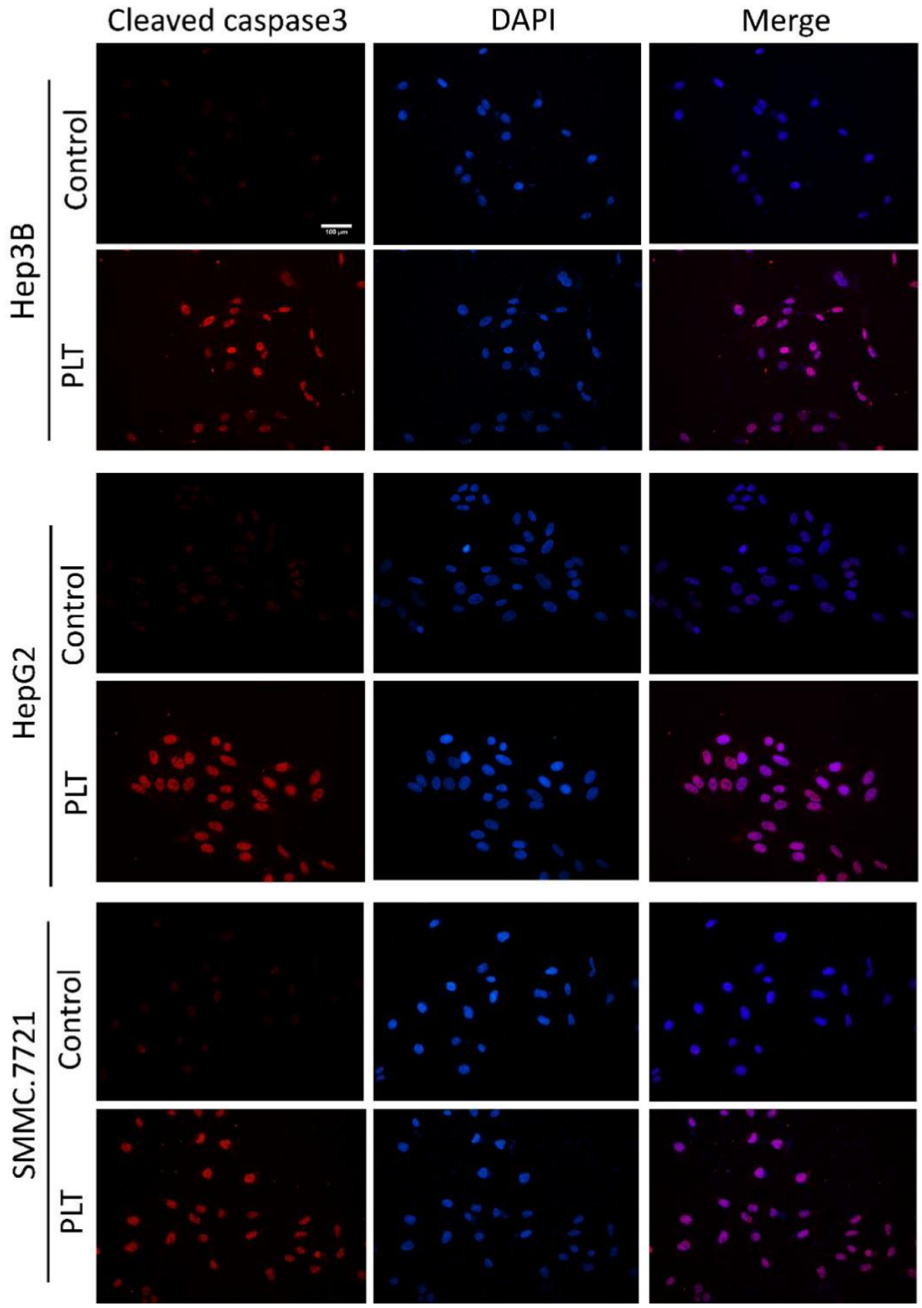

Supplementary Fig. 7. Recruited platelets increase the level of cleaved caspase 3 in HCC cells. In CM-induced platelet migration assay, platelets in the lower chamber were collected and then cultured with HCC cells for 24 hours. Analysis of cleaved caspase 3 expression by immunofluorescence (n=3). Cleaved caspase 3: red. Scar bar: 100μm.
